# Lifestyle, health characteristics and alcohol abuse in young adults who are non-daily smokers

**DOI:** 10.1590/S1516-31802010000600008

**Published:** 2010-12-02

**Authors:** Maria Izabel de Ugalde Marques da Rocha, Juan Pablo Barrio-Lera, Gabriel Behr Gomes Jardim, Amanda Brondani Mucellini, Luiza Cirolini, Ivo Emilio da Cruz Jung, Maria Fernanda Mânica-Cattani, Aron Ferreira da Silveira, Olmiro Cezimbra de Souza, Ivana Beatrice Mânica da Cruz

**Affiliations:** I MSc, PhD. Professor of Morphology, Department of Morphology, Health Science Center, Universidade Federal de Santa Maria (UFSM), Santa Maria, Rio Grande do Sul, Brazil.; II MSc, PhD. Professor of Physiology, Postdoctoral Program on Biomedicine, Department of Biomedicine, Universidad de León (UNILEON), Spain.; III Undergraduate medical student, Universidade Federal de Santa Maria (UFSM), Santa Maria, Rio Grande do Sul, Brazil.; IV Undergraduate biology student, Universidade Federal de Santa Maria (UFSM), Santa Maria, Rio Grande do Sul, Brazil.; V Undergraduate psychology student, Centro Universitário Franciscano (UNIFRA), Santa Maria, Rio Grande do Sul, Brazil.; VI Postgraduate student of Toxicological Biochemistry, Universidade Federal de Santa Maria (UFSM), Santa Maria, Rio Grande do Sul, Brazil.; VII MD, PhD. Professor of Morphology, Department of Morphology, Health Science Center, Universidade Federal de Santa Maria (UFSM), Santa Maria, Rio Grande do Sul, Brazil.; VIII MSc. Professor of Education, Department of Morphology, Health Science Center, Universidade Federal de Santa Maria (UFSM), Santa Maria, Rio Grande do Sul, Brazil.; IX MSc, PhD. Professor of Genetics and Molecular Biology, Universidade Federal de Santa Maria (UFSM), Santa Maria, Rio Grande do Sul, Brazil.

**Keywords:** Smoking, Young adult, Alcoholism, Life style, Health, Tabagismo, Adulto jovem, Alcoolismo, Estilo de vida, Saúde

## Abstract

**CONTEXT AND OBJECTIVES:**

Despite the decline in the prevalence of tobacco use in many countries, including Brazil, there are growing numbers of smokers who continue to smoke at a low daily rate, or less frequently (non-daily smokers). This group needs to be better characterized in order to direct preventive actions and public health policies. The aim here was to compare lifestyle, health characteristics and alcoholism problems among young adult smokers, non-daily smokers and non-smokers.

**DESIGN AND SETTING:**

This was a cross-sectional study in which volunteers from the university community and its surrounds in Santa Maria, State of Rio Grande do Sul, Brazil, were included between October 2007 and January 2008.

**METHODS:**

Out of 1240 volunteers initially contacted in a university cafeteria, a total of 728 participants of mean age 22.45 ± 3.32 years were selected for final analysis. Data were collected using structured questionnaires.

**RESULTS:**

In general, it was observed that the non-daily smokers showed intermediate characteristics in relation to the smokers and non-smokers. However, there was a significant association between non-daily smoking and alcohol abuse. The non-daily smokers presented an odds ratio of 2.4 (95% confidence interval: 1.10-5.48) in relation to the daily smokers and an odds ratio of 3.3 (confidence interval: 1.7-6.5) in relation to the non-smokers, with regard to presenting a positive CAGE test, thereby indicating alcohol abuse or dependence.

**CONCLUSION:**

The study suggested that non-daily smoking and alcohol consumption were concomitant behaviors.

## RESUMO

**CONTEXTO E OBJETIVO:**

Apesar do declínio na prevalência do tabagismo em diversos países, incluindo o Brasil, existe um número crescente de fumantes que continua a fumar em baixa quantidade diária ou com menor frequência (fumantes esporádicos). Esse grupo precisa ser mais bem caracterizado para que se possa direcionar ações preventivas e políticas de saúde pública. O objetivo foi comparar o estilo de vida, características de saúde e problemas de alcoolismo entre fumantes, fumantes esporádicos e não fumantes entre adultos jovens.

**TIPO DE ESTUDO E LOCAL:**

Estudo transversal em que se incluíram voluntários da comunidade universitária e do seu entorno entre outubro de 2007 a janeiro de 2008 em Santa Maria, Rio Grande do Sul, Brasil.

**MÉTODOS:**

A partir de um contato inicial com 1.240 voluntários numa cafeteria universitária, 728 participantes com idade média de 22.45 ± 3.32 anos foram selecionados para análise final. Os dados foram coletados através de um questionário estruturado.

**RESULTADOS:**

Em geral, foi observado que os fumantes esporádicos apresentaram características intermediárias em relação aos fumantes e não fumantes. Entretanto, foi encontrada uma associação significativa entre fumo esporádico e abuso de álcool. Os fumantes esporádicos apresentaram uma razão de chance de 2,4 vezes (intervalo de confiança de 1,10-5,48) em relação aos fumantes diários e uma razão de chance de 3,3 (intervalo de confiança de 1,7-6,5) em relação aos não fumantes de apresentar um teste CAGE positivo indicador de abuso ou dependência de álcool.

**CONCLUSÃO:**

O estudo sugere que o tabagismo esporádico e o consumo de álcool são comportamentos concomitantes.

## INTRODUCTION

Tobacco use is one of the leading preventable causes of premature death, disease and disability around the world.^1^ Despite the decline in the prevalence of tobacco use in many populations, including Brazil (where smoking prevalence dropped from 36.5% in 1989 to 24% in 2002),^2^ there are growing numbers of smokers who continue to smoke at a lower rate, either daily or less frequently. This group has been described as “low-rate smokers,^3^ “chippers”,^4^ “social smokers”^5^ and non-daily or occasional smokers. Studies conducted in the last decade have suggested that non-daily smokers are a growing subpopulation of smokers.^6^

A large survey that included 36,060 smokers analyzed the prevalence of non-daily smokers and found that this group made up a substantial segment of the smoking population who were not just beginning to smoke or trying to quit. Many had developed a long-standing pattern of non-daily smoking, smoking relatively few cigarettes on the days when they did smoke.^7^ An investigation analyzing the American subjects of the National Health Interview Survey reported that non-daily smokers showed better health than did daily smokers but had some health status indicators suggesting worse health than among non-smokers.^8^ Although researchers generally agree on what constitutes a non-smoker (i.e. someone who has never smoked) or a daily smoker (i.e. someone who smokes every day or almost every day), considerable variation exists in the definitions for occasional smokers. In general, studies combine non-daily smokers in the same category as daily smokers.^9,10^ The problem in mixing these two categories is that some investigations on the non-daily smoker group suggest that occasional smoking may be a stable pattern for long periods and may correlate with other lifestyle indicators such as alcohol use.^4^

Recently, Harrison and McKee^11^ investigated binge drinking and non-daily cigarette smoking among 40 young adults, and the results obtained showed that non-daily smokers often use alcohol and cigarettes at the same time. The authors commented that because of the strong relationship between non-daily smoking and binge drinking, non-daily smoking is of considerable public health concern.

Although smoking prevalence has decreased in Brazil, it is still a matter of great public health concern. Data from the Brazilian National Cancer Institute (Instituto Nacional de Câncer, INCA) estimate that the annual number of smoking-related deaths is 200,000.^12^ Surprisingly, cities located in more developed regions such as the States of Rio de Janeiro, São Paulo and Rio Grande do Sul show higher incidence of smoking than do those in less developed regions. Since epidemiological studies on non-daily smoking young adults are still at an initial stage in the Brazilian population, a general study was designed here, in order to determine whether there are any differences in general lifestyle, health characteristics and alcohol problems between non-daily smokers, daily smokers and non-smokers.

## OBJECTIVES

To determine whether there are any differences in general lifestyle, health characteristics and alcohol problems between non-daily smokers, daily smokers and non-smokers.

## METHODS

This cross-sectional study was part of the Southern Brazil GENESIS Project, which investigated genetic-environmental interactions in healthy aging and age-related diseases. The first phase of the project included several investigations in a free-living elderly community (≥ 60 years old) between 2000 and 2006, including associations between gene polymorphism and several risk factors for chronic diseases^13,14^ and smoking addiction.^15^

This study will be combined with future complementary investigations using genetic approaches, and therefore all the subjects included were Caucasian, given that the State of Rio Grande do Sul in Brazil has higher prevalence of this ethnic group than of other groups due to the massive European colonization.

During the second phase, which included this investigation, data were gathered from a young adult population in order to conduct a study that took an integrative approach towards the dynamic process associated with smoking addiction. In the cross-sectional investigation described here, the subjects included were recruited from students, professors and visitors in a university community (Universidade Federal de Santa Maria, Santa Maria, State of Rio Grande do Sul, Brazil) between October 2007 and January 2008. Previously, trained research assistants made the first contact with the respondents in the university cafeteria, where they were invited to participate as volunteers. Each researcher assistant interviewed 24 volunters. A total of 1240 participants aged 22.45 ± 3.32 years (range: 18-32 years) were enrolled in the study. Employees with socioeconomic and cultural characteristics and ages that were much different from those from the young students (since these can influence the prevalence of smoking addiction) and those who did not complete the questionnaire were excluded from the final analysis. Therefore, the total number of subjects studied was 728.

The smoking groups were defined using the following criteria: daily and non-daily smokers needed to report having smoked at least 100 cigarettes over their lifetimes and to be currently smoking cigarettes on some days (non-daily smokers) or every day (daily smokers). One hundred and eighteen participants were classified as daily smokers, 48 as non-daily smokers, 23 as former smokers (≥ two years without smoking) and 562 as non-smokers. Evans et al.^16^ found that the proportion of current non-daily smokers stabilized around three years after initiation of fairly regular smoking. These authors recommended that five years should be used as a conservative estimate. This question was considered in the smoking classification and it was found that 82 daily smokers (77%) and 36 non-daily smokers (76.5%) presented a stable smoking pattern (≥ five years). Since the sample investigated was very young, it was not known whether these subjects would go back to smoking, so it was decided to exclude this category from the analysis in order to avoid possible bias.

The subjects were briefed about the purpose of the study and informed that participation was voluntary. They were asked to answer all questions honestly, and were reassured about the anonymity and confidentiality of the information. The survey took 20-25 minutes for them to complete. The data were gathered using a structured questionnaire that was presented by the investigators, who gave assistance for filling it in.

The questionnaire contained questions relating to self-reported weight, height, lifestyle (food and physical activity behavior), health conditions (presence of morbidities relating to cardiovascular and respiratory diseases and tobacco use: obesity, dyslipidemia, diabetes type II, hypertension and asthma), mental conditions (depression, anxiety and stress) and use of medications relating to sleep and attention disorders. The weight and height information were used to calculate the body mass index (kg/m^2^) and to classify the subjects as overweight (> 25 kg/m^2^) or obese (> 30 kg/m^2^).

The Fagerstrom Test for Nicotine Dependence was used to measure the severity of nicotine dependence among daily smokers. It was observed that in our sample of daily smokers, 53 (62.9%) showed low nicotine dependence, 22 (19.4%) moderate dependence and 21 (17.7%) strong dependence. Additionally, the four-item CAGE questionnaire^17^ for evaluating alcohol abuse and dependence was applied to all subjects.

Statistical analysis was performed using version 16.0 of the Statistical Package for the Social Sciences (SPSS) software (version 15.0). Tests on associations between categorical variables and the three groups were performed using a univariate nonparametric chi-square test for independent samples. Multivariate analysis was performed using logistic regression (backward Wald method), including all variables with univariate statistical significance of P < 0.1. Quantitative variables were analyzed using one-way analysis of variance (ANOVA) followed by the post-hoc Bonferroni test. All analyses were two-tailed and the null hypothesis was rejected when P < 0.05

The Ethics Committee of Universidade Federal de Santa Maria approved the study protocol. Subjects were informed that they had been selected and were asked to attend the university's health center to be enrolled in the study. Verbal consent was obtained from the participating volunteers, and the investigators spoke to the students in an informal setting.

## RESULTS

The demographic variables were similar among the three groups, whereas lifestyle and health indicators showed some important variations, as shown in Table 1. The lifestyle indicators (food and physical activity behavior) and physical health indicators among daily smokers, non-daily smokers and non-smokers, which are also presented in Table 1, showed an intermediate pattern for non-daily smokers, in relation to daily smokers and non-smokers.

**Table 1.** Demographic, lifestyle and health characteristics that were self-reported by subjects classified as daily smokers, non-daily smokers and non-smokers

Variable		Groups			P
		Daily smokers	Non-smokers	Non-daily smokers	
Gender	Male n (%)	66 (18.0)	271 (73.8)	30 (8.2)	0.079
	Female n (%)	52 (14.4)	291 (80.6)	18 (5.0)	
Age (mean ± SD)		22.8 ± 3.4^a^	22.3 ± 3.3^a^	23.08 ± 3.6^a^	0.177
Undergraduate/graduate students n (%)		75 (63.6)^a^	413 (73.5)^a^	36 (75.0)^a^	0.327
Education (≥ 12 years) n (%)		73 (61.9)^a^	367 (65.3)^a^	32 (66.7)^a^	0.748
Having breakfast (all days) n (%)		37 (34.9)^a^	338 (58.9)^b^	16 (33.3)^a^	0.000
Fruit/vegetables intake (≥ 3 days/week) n (%)		33 (31.1)^a^	104 (18.4)^b^	10 (20.8)^b^	0.037
Sweet/snack consumption (≥ 5 days/week) n (%)		42 (39.6)^a^	218 (38.0)^a^	23 (47.9)^a^	0.064
Coffee (> 5 days/week) n (%)		35 (33.0)^a^	178 (31.0)^a^	12 (25.0)^a^	0.878
Tea and “chimarrão” beverages (≥ 5 days/week) n (%)		43 (40.6)^a^	158 (27.6)^b^	16 (33.3)^b^	0.022
Regular exercises (≥ 3 days/week) n (%)		42 (39.6)^a^	299 (52.1)^b^	26 (54.2)^b^	0.050
Overweight (> 25 kg/m^2^)		38 (35.8)^a^	101 (17.6)^b^	07 (14.9)^b^	0.000
Hypertension		06 (5.7)^a^	07 (1.2)^b^	2 (4.2)^ab^	0.007
Dyslipidemia		04 (3.8)^a^	23 (4.0)^a^	02 (4.2)^a^	0.991
Asthma		18 (17.0)^a^	81 (14.1)^a^	09 (18.8)^a^	0.547
Sleep medications		19 (17.9)^a^	33 (5.7)^b^	03 (6.3)^b^	0.000
Attention deficit medications		05 (4.7)^a^	35 (6.1)^a^	05 (10.4)^a^	0.390

n = sample size; SD = standard deviation; the small different letters in the table, in the comparison of the smoker groups, indicate statistical differences among them (P < 0.05) evaluated by one-way variance analysis followed by Bonferroni post hoc test. Except for age and the education variables, which were compared by means of one-way analysis of variance (ANOVA) followed by the Bonferroni *post hoc* test, the other variables were compared using the chi-square test.

The daily and non-daily smokers reported similar lower frequencies with regard to having breakfast every morning, compared with non-smokers. However, for the other lifestyle variables, the non-daily smokers showed more similarities with the non-smoker group. The non-daily and non-smoker groups reported eating fruits and vegetables ≥ three times per week and engaging in physical activity ≥ three times per week (more than daily smokers). Coffee intake was similar in the three groups, whereas the daily smokers reported drinking several types of tea or an infusion popularly known as “mate” or “chimarrão” (*Ilex paraguariensis*), more frequently than the non-daily smokers and non-smokers did. Chimarrão is a traditional tea-like brew widely enjoyed in Argentina, Paraguay, Uruguay and southern Brazil, which is prepared using dried green leaves steeped in hot water, in a vessel called a “cuia.”

The prevalences of overweight, hypertension and sleep medication use were similar among non-daily and non-smokers and statistically higher among daily smokers, as shown in Table 1.

Alcohol abuse, family histories of smoking, depression, anxiety and stress that were self-reported by subjects classified as daily smokers, non-daily smokers and non-smokers are presented in Table 2. The non-daily smokers had higher prevalence of positive findings from the CAGE test for alcohol abuse, compared with daily smokers and non-smokers.

**Table 2.** Alcohol abuse, family history of smoking, depression, anxiety and stress that were self-reported by subjects who were classified as daily smokers, non-daily smokers or nonsmokers

Variable	Groups			P
	Daily smokers n (%)	Non-smokers n (%)	Non-daily smokers n (%)	
Alcohol abuse/dependence (two positive responses in CAGE test)	15 (14.2)^a^	64 (11.1)^a^	14 (29.2)^b^	0.001
Self-reported depression	30 (28.3)^a^	90 (15.7)^b^	09 (18.8)^b^	0.007
Self-reported anxiety	48 (45.3)^a^	144 (25.1)^b^	11 (22.9)^b^	0.000
Self-reported stress	33 (31.1)^a^	136 (23.7)^a^	10 (20.8)^a^	0.216
Family history of smoking				
≥ 2 parents, grandparents or siblings who were smokers	75 (63.6)^a^	278 (49.5)^b^	25 (52.1)^b^	0.021
Father and mother were smokers	12 (11.9)^a^	22 (4.6)^a^	03 (7.3)^a^	0.061
At least one parent was a smoker	54 (53.5)^a^	202 (42.3)^a^	15 (36.6)^a^	0.078
Living with smokers n (%)	58 (50.4)^a^	42 (11.3)^b^	10 (25.0)^c^	0.001

n = sample size; the small different letters in the table, in the comparison of the smoker groups, indicate statistical differences among them (P < 0.05) evaluated by one-way variance analysis followed by Bonferroni post hoc test.

However, the self-reported prevalences of depression, anxiety and family history and the percentage of subjects who lived with smokers were statistically similar between non-daily smokers and non-smokers. In this regard, there was higher prevalence of these characteristics among daily smokers.

An additional comparison showed that the non-daily and daily smokers started smoking at similar ages (non-daily smokers = 15.1 ± 2.3 years old and daily smokers = 16.09 ± 2.9) (P = 0.187). When asked about attempts to quit smoking, the non-daily smokers tried to quit fewer times than daily smokers did. In this regard, only 27.6% of the daily smokers had never tried to quit smoking, whereas 52.9% of the non-daily smokers had never tried to quit (P = 0.023).

Because the non-daily smokers showed some variables that were similar to daily smokers and others that were similar to non-smokers, a second statistical multivariate analysis was performed to compare the characteristics in just two smoker categories that showed significant differences in the first analysis. In this second comparison, gender influence was also evaluated. The variables that remained independent of associations with daily smokers were anxiety indicators, living with other smokers and alcohol abuse/dependence (CAGE) (Table 3). The non-daily smokers showed an odds ratio of 2.4 (95% confidence interval, CI: 1.10-5.48) of anxiety risk when compared with the daily smokers with a positive CAGE test; and showed an odds ratio of 3.3 (95% CI: 1.7-6.5), compared with the non-smokers.

**Table 3.** Multivariate comparison between daily smokers and non-daily smokers

Variables	p	Odds ratio	95% confidence interval
Living with smokers	0.026	2.566	1.121-5.871
Anxiety indication	0.049	2.261	1.10-532
Alcohol dependence/abuse (two positive responses in CAGE tests)	0.021	0.354	0.146-0.856

## DISCUSSION

In this study, different lifestyles and health variables among young Brazilian adults who were daily smokers, non-daily smokers and non-smokers were compared, and the results showed that non-daily smokers presented intermediate characteristics in relation to daily smokers and non-smokers. Unfortunately, no similar previous Brazilian studies on non-daily smoking prevalence could be found for comparison purposes. Thus, this present investigation may be considered to be the first to take this group into account. These results are similar to the findings from the United States National Health Interview Survey,^18^ which reported that non-daily smokers showed better health than daily smokers did, but had some health status indicators suggesting a worse health situation than among non-smokers. Since the sample analyzed here was composed of young adults who had a lower incidence of chronic disorders, the main variable that needs to be considered is a possible association between non-daily smoking and alcohol abuse/dependence, as measured using the CAGE test.

This result is intriguing, since several studies have suggested that higher nicotine dependence is associated with increased alcohol consumption.^19,20^ This premise is corroborated by a study conducted in Rio Grande do Sul (the same region as where the present study was conducted), which also applied the CAGE questionnaire to a general population sample (1397 adults aged ≥ 35 years) and observed a statistical association between smoking and alcoholism. Alcoholism was associated with smoking (67% of the alcoholics and 44% of the non-alcoholics reported having a smoking habit), low income and rudimentary cultural and professional levels.^21^ Therefore, it would be expected to find higher prevalence of alcohol abuse among daily smokers.

Epidemiological studies in developed countries such as the United States have suggested that non-daily smokers have higher rates of binge drinking of alcohol and related conditions, compared with non-smokers and daily smokers.^22^ Other investigations have also described an association between alcohol use and daily/non-daily smoking, compared with not smoking.^23^ Although the present study did not make an analysis on binge drinking of alcohol and related conditions, in relation to this sample, the data obtained from the CAGE test indicated that the non-daily smoker group presented greater use of alcohol than daily smokers did. Based on these data, complementary studies need to be performed in order to understand the extent of the association between non-daily smoking and drinking patterns.

Non-daily smokers could have a “shared tobacco-alcohol addiction”, with binge peaks during particular periods or situations. Harrison and McKee^24^ analyzed the patterns of alcohol and cigarette use among young non-daily smokers. The volunteers reported that they consumed 7.5 ± 3.6 drinks and 3.3 ± 2.5 cigarettes over the course of 5.15 hours, and that almost all of them started smoking by the second hour, when they had already consumed 2.87 ± 1.62 drinks. The results suggest that non-daily smokers often concurrently use alcohol and cigarettes, thus corroborating the suggestion that non-daily smokers are a differential category.

This study presents certain limitations, including the use of the CAGE questionnaire as indicative of alcohol abuse/dependence. This instrument is one of the most widely used alcohol screening instruments and has high sensitivity and specificity for identifying patients with alcohol dependence. Although the CAGE questionnaire has proven to be a valid instrument for screening alcohol-dependence problems in clinical populations, it is less effective in young populations.^25,26^ Therefore, the CAGE questionnaire sometimes fails to identify young people who are drinking heavily or who have alcohol-related problems but do not yet show symptoms of alcohol dependence. Bühler et al.^26^ analyzed the role of age, gender and drinking patterns in relation to inconsistent identification of alcohol-related problems using CAGE and the criteria of the Diagnostic and Statistical Manual of Mental Disorders, fourth edition (DSM-IV), and they found that the classification using these instruments varies according to gender, age and drinking pattern.

However, in this study, alcohol abuse/dependence measured using the CAGE questionnaire was compared between three smoking categories (daily, non-daily and non-smokers) and, for this reason, the limitations of this instrument could be minimized. Recent studies have also applied the CAGE questionnaire to surveys among young adults, as performed by Bewick et al.^27^ These authors used a randomized controlled trial to establish that an electronic web-based personalized feedback intervention was effective. Their study on 506 volunteers considered gender, age and schooling level, and used the CAGE score as a screening tool for alcohol use disorders. They found from a pre-survey analysis that 279 participants (55%) reported potentially problematic alcohol consumption (i.e. scoring positively in two or more CAGE items). This frequency was almost twice as high as found in our sample.

The data presented here corroborate previous studies that considered non-daily smoking to be a stable pattern of smoking behavior, rather than as a transitional phase, either between initiation and daily smoking or between regular smoking and cessation. Unfortunately, it is not known whether this type of smoking behavior is a response to increased tobacco control measures and increased public health messages regarding the health risks of smoking, or whether this was always there but not perceived until now. The data presented here do not suggest that non-daily smokers plan to quit smoking. However, it is not known whether this smoker group believes that it needs to consider quitting.

Morley et al.^22^ commented that identification of this smoker category raises two important questions regarding research on the impact of public health interventions on smoking. Firstly, it needs to be determined how smoking bans in pubs and clubs will affect the proportion of smokers who are occasional or social smokers. Secondly, it needs to be asked whether increasing social disapproval of smoking will make people less likely to disclose their smoking behavior and thus affect the reliability of self-reported measurements of smoking, which form the basis for planning and assessing the impact of tobacco control initiatives. From the results described here, a further question that needs to be addressed emerges: are neurophysiological addiction pathways common to several drugs, including alcohol and smoking? Thus, if non-daily smokers show intermediate patterns between daily smokers and non-smokers, it needs to be determined what possible neurological and behavioral differences or similarities they might show in relation to daily smokers and non-smokers.

## CONCLUSIONS

In conclusion, this study found an intermediate lifestyle and health pattern among stable non-daily smokers, compared with daily smokers and non-smokers. This corroborates the idea that this is a differential category that is probably closely associated with alcohol abuse. Additional investigations on the possible association with non-daily smoking and other drug and non-drug addictive behaviors (including electronic games), along with the associated biological, environmental and social causes, are a challenge that needs to be understood so that, in the future, the addictive behavior of non-daily smoking can be controlled.

## References

[1] Ezzati M, Henley SJ, Thun MJ, Lopez AD (2005). Role of smoking in global and regional cardiovascular mortality. Circulation.

[2] World Health Organization (1997). Tobacco or health: a global status report.

[3] Owen N, Kent P, Wakefield M, Roberts L (1995). Low-rate smokers. Prev Med.

[4] Shiffman S, Fischer LB, Zettler-Segal M, Benowitz NL (1990). Nicotine exposure among nondependent smokers. Arch Gen Psychiatry.

[5] Gilpin E, Cavin SW, Pierce JP (1997). Adult smokers who do not smoke daily. Addiction.

[6] Shiffman S (1989). Tobacco “chippers”--individual differences in tobacco dependence. Psychopharmacology (Berl).

[7] Hassmiller KM, Warner KE, Mendez D, Levy DT, Romano E (2003). Nondaily smokers: who are they?. Am J Public Health.

[8] Tong EK, Ong MK, Vittinghoff E, Pérez-Stable EJ (2006). Nondaily smokers should be asked and advised to quit. Am J Prev Med.

[9] Mayhew KP, Flay BR, Mott JA (2000). Stages in the development of adolescent smoking. Drug Alcohol Depend.

[10] Oksuz E, Mutlu ET, Malhan S (2007). Characteristics of daily and occasional smoking among youths. Public Health.

[11] Harrison EL, McKee SA (2008). Young adult non-daily smokers: patterns of alcohol and cigarette use. Addict Behav.

[12] Rodrigues GA, Galvão V, Viegas CA (2008). Prevalência do tabagismo entre dentistas do Distrito Federal [Prevalence of smoking among dentists in the Federal District of Brasília, Brazil]. J Bras Pneumol.

[13] Da Cruz IB, Oliveira G, Taufer M (2003). Angiotensin I-converting enzyme gene polymorphism in two ethnic groups living in Brazil's southern region: association with age. J Gerontol A Biol Sci Med Sci.

[14] Taufer M, Peres A, de Andrade VM (2005). Is the Val16Ala manganese superoxide dismutase polymorphism associated with the aging process. J Gerontol A Biol Sci Med Sci.

[15] Prado-Lima PS, Cruz IB, Schwanke CH, Netto CA, Licinio J (2006). Human food preferences are associated with a 5-HT(2A) serotonergic receptor polymorphism. Mol Psychiatry.

[16] Evans NJ, Gilpin E, Pierce JP (1992). Occasional smoking among adults: evidence from the California Tobacco Survey. Tob Control.

[17] Ewing JA (1984). Detecting alcoholism. The CAGE questionnaire. JAMA.

[18] DiNicola AF, Seltzer DM (2009). National Health Interview Survey on the United States smokers. Mil Med.

[19] Batel P, Pessione F, Maitre C, Rueff B (1995). Relationship between alcohol and tobacco dependencies among alcoholics who smoke. Addiction.

[20] Toneatto A, Sobell LC, Sobell MB, Kozlowski LT (1995). Effect of cigarette smoking on alcohol treatment outcome. J Subst Abuse.

[21] Chaieb JA, Vitola D, Silva MS (1984). An epidemiologic survey of smoking patterns and chronic obstructive bronchopulmonary disease in Porto Alegre, Brazil. Bull Pan Am Health Organ.

[22] Morley KI, Hall WD, Hausdorf K, Owen N (2006). ‘Occasional’ and ‘social’ smokers: potential target groups for smoking cessation campaigns?. Aust N Z J Public Health.

[23] McKee SA, Hinson R, Rounsaville D, Petrelli P (2004). Survey of subjective effects of smoking while drinking among college students. Nicotine Tob Res.

[24] Heck EJ, Williams MD (1995). Using the CAGE to screen for drinking-related problems in college students. J Stud Alcohol.

[25] O'Hare T, Tran TV (1997). Predicting problem drinking in college students: gender differences and the CAGE questionnaire. Addict Behav.

[26] Bühler A, Kraus L, Augustin R, Kramer S (2004). Screening for alcohol-related problems in the general population using CAGE and DSM-IV: characteristics of congruently and incongruently identified participants. Addict Behav.

[27] Bewick BM, Trusler K, Mulhern B, Barkham M, Hill AJ (2008). The feasibility and effectiveness of a web-based personalised feedback and social norms alcohol intervention in UK university students: a randomised controlled trial. Addict Behav.

